# Malaria vaccine research & innovation: the intersection of IA2030 and zero malaria

**DOI:** 10.1038/s41541-020-00259-3

**Published:** 2020-11-20

**Authors:** David C. Kaslow

**Affiliations:** PATH, 2201 Westlake Avenue, Suite 200, Seattle, WA 98121 USA

**Keywords:** Vaccines, Infectious diseases

This collection of malaria vaccine research and innovation papers highlights the intersection of efforts to: (1) achieve the pan-African^1^ and global goal^2^ of “Zero Malaria”; and (2) make the compelling case for immunization^3^ as a set of new tools for malaria control and elimination.

## Problem and opportunity statement

Despite a ~30% reduction in clinical cases and ~60% reduction in lives lost from *Plasmodium ssp*. infections over the past two decades, malaria continues to relentlessly sap the well-being of an estimated 228 million people worldwide (95% confidence interval [CI]: 206–258 million) and result in the global demise of an estimated 405,000 persons annually. The effort, supported by a US$ 2.7–3.2 billion annual investment, to “bend the curve” towards zero malaria by 2030 has stalled over the past five years^4^. This plateau is reminiscent of the decades-long effort to break through the ~80% ceiling on reaching the goal of fully immunizing every child worldwide. Despite immunization of 116 million children annually, 20 million infants fail to receive a full course of essential vaccines and 13 million infants receive no vaccines whatsoever—these “zero dose” children are highlighted in the Immunization Agenda 2030^3^ (IA2030), recently adopted by the World Health Assembly^5^. The shared state of stalled progress towards equity and coverage of effective malaria interventions and essential vaccines has a common solution—new tools.

The early promise of innovative vector control and single-dose radical cure for malaria have yet to deliver the impact needed to achieve “zero malaria”. That said, steadily over the last two decades, as reviewed by Duffy and Gorres, a pipeline of vaccine candidates against the most lethal human malaria, *P. falciparum*, and the most prevalent, *P. vivax*, has been assembled^6^. Novel vaccine platforms (e.g., *P. berghei* sporozoite-based human vaccine candidates^7^), better defined correlates of protection in non-human primates^8^, and orthogonal learnings from other mosquito-transmitted pathogens (e.g., West Nile Virus)^9^ portend an even more robust pipeline of needed new tools, if adequate investments are made.

## *P. falciparum* vaccine development stage-by-stage and step-by-step

Malaria vaccine targets are typically assigned to one of three sequential stages of the parasite’s lifecycle: blood stage—through which the parasite causes all human pathology; sexual and sporogony stage—through which the parasite is transmitted from human host to female mosquito vector; and, pre-erythrocytic stage—through which the parasite infects humans during a subsequent bloodmeal by an infected mosquito (see Fig. 1, ref. ^6^). Despite decades of efforts directed against targets throughout the lifecycle, only one vaccine candidate, the pre-erythrocytic circumsporozoite (CSP)-based RTS,S/AS01_E_, has advanced through licensure and pilot implementation^6^. A myriad of biological and technical barriers have been encountered; however, step-by-step, these barriers have been chipped away. Advances include: new insights into the evolution of the immune response to the major pre-erythrocytic target, CSP^10^, and into the role of complement-fixing antibodies in blood stage clinical immunity^11^; the ability to express and manufacture full length versions of blood stage targets, e.g., the highly promising, highly conserved reticulocyte-binding protein homolog 5 (PfRH5)^12^, as well as the 196-kDa merozoite surface protein 1 (the primary structure first described by Holder, et al. in 1985^13^), the latter of which demonstrated favorable safety and immunogenicity in a first-in human study^14^; and, the delivery of target malaria parasite antigens by measles vectors to overcome the barrier of waning immune responses, resulting in durable memory and protection, at least in a murine model^15^.

To be clear, significant challenges remain—for example, immune interference when concomitantly administering other vaccines, such as BCG for tuberculosis^16^, or administering multi-stage targets by multiple vaccine platforms, as observed with an adjuvanted virus-like particle, RTS,S/AS01_B_, and viral-vectors expressing the multiple-epitope thrombospondin-related adhesion protein (ME-TRAP)^17^. That said, molecular approaches to identify and direct immune responses to specific promising epitopes^18^ and use of novel particle-forming lipid-based adjuvants^19^ provide paths forward for poorly immunogenic targets, including those designed to interrupt transmission from human to mosquito.

## Malaria vaccine use cases—pregnancy malaria must not be left behind

The longstanding mindset that vaccine evaluation during pregnancy should be delayed to post-licensure studies is obsolete. Even when pregnant women and their offspring do not have a higher risk of disease, there is an ethical rationale to evaluate at least vaccine safety pre-licensure^20^. Pregnancy malaria presents an even clearer case for prioritizing vaccine development as both mother and offspring have a well-described higher risk of disease^6^. More than two decades ago, antibodies to VAR2CSA, a member of the *P. falciparum* erythrocyte membrane protein 1 (PfEMP1) family, were associated with protection, identifying VAR2CSA as a promising vaccine target^21^. Placenta malaria vaccine (PMVs) research and innovation continues to progress^6^, including through the development of new animal models^22^ and down-selection of lead PVM candidates^23^.

## *P. vivax*—the other malaria

Despite being the most prevalent human malaria parasite, investments in *P. vivax* vaccine development have been significantly smaller than that in the more lethal, albeit similarly morbid, falciparum cousin. Many *P. falciparum* vaccine targets have homologs in *P. vivax*^6^, so a fast follow-on vivax malaria vaccines based on safe, effective, affordable falciparum malaria vaccines have a reasonable, high likelihood of success. That said, several distinct differences in *P. vivax* biology, such as liver stage hypnozoites and rapid development of sexual stages directly from liver schizonts, require a *P. vivax*-specific vaccine research and innovation strategy. A critical differential feature of blood-stage vivax parasites is the use of Duffy antigen receptor for chemokines (DARCs) on human reticulocytes as a major invasion pathway. Recent insights into the structural basis of anti-*P. vivax* Duffy Binding Domain (PvDBP) immunity^24,25^ and early clinical results from a PvDBP vaccine candidate^26^ are encouraging; however, given the parasite’s gene amplification mechanisms to evade anti-PvDBP immunity^27^, developing a blood stage vivax malaria vaccine won’t be trivial.

## Research and Innovation as a driver for creating a compelling value proposition for malaria vaccines

The promising *P. falciparum* and *P. vivax* vaccine pipeline faces a significant resource shortfall as candidates head into late-stage development—this increasingly more apparent resource gap, or second “Valley of Death”, is also faced by most, if not all, late stage vaccine candidates for pathogens afflicting primarily those living in low resource settings^28^. Hopefully, the recent global adoption of the Research and Innovation pillar of IA2030 and the growing African continent-led commitment to zero malaria will lead to the investments needed to generate the evidence that support the compelling value proposition required to build bridges for promising malaria vaccine candidates to become affordable, effective, sustainable new tools—part of the solution to regain the prior trajectory towards zero malaria.
